# Efficacy and tolerability of fixed-dose amlodipine/olmesartan medoxomil with or without hydrochlorothiazide in Hispanic and non-Hispanic patients whose blood pressure is uncontrolled on antihypertensive monotherapy

**DOI:** 10.1177/1753944712452190

**Published:** 2012-08

**Authors:** Henry Punzi, Ali Shojaee, Jen-Fue Maa

**Affiliations:** Punzi Medical Center, 1932 Walnut Plaza, Carrollton, TX 75006, USA; Daiichi Sankyo, Inc., Parsippany, NJ, USA; Daiichi Sankyo, Inc., Parsippany, NJ, USA

**Keywords:** 24 h blood pressure control, ambulatory blood pressure, amlodipine, fixed-dose combination, Hispanic, hydrochlorothiazide, hypertension, olmesartan medoxomil

## Abstract

**Objectives:**

This is a prespecified subgroup analysis in Hispanic and non-Hispanic patients of a study that evaluated blood pressure (BP) control with fixed-dose amlodipine/olmesartan medoxomil (AML/OM)-based therapy in patients whose condition was uncontrolled on prior monotherapy.

**Methods:**

In this prospective, open-label, dose-titration study, patients with uncontrolled BP after at least 1 month of antihypertensive monotherapy were switched to fixed-dose AML/OM 5/20 mg. Patients were uptitrated to AML/OM 5/40 and 10/40 mg, with uptitration to AML/OM + hydrochlorothiazide 10/40 + 12.5 mg and 10/40 + 25 mg to achieve target BP. The primary efficacy endpoint was the cumulative proportion of patients achieving seated cuff systolic BP (SeSBP) less than 140 mmHg (<130 mmHg in patients with diabetes mellitus) at 12 weeks. Secondary endpoints included SeBP goal rates, ambulatory BP (ABP) target rates, and mean change from baseline in seated cuff BP (SeBP) and ABP at weeks 12 and 20.

**Results:**

Mean baseline BP was similar in Hispanics (153.6/92.8 mmHg; *n* = 105) and non-Hispanics (153.7/91.8 mmHg; *n* = 894). At 12 weeks, 72.0% of Hispanics and 76.3% of non-Hispanics achieved the primary endpoint. At week 12, goal rates for cumulative SeBP (<140/90 mmHg or <130/80 mmHg in patients with diabetes) were 69.0% and 71.5% in Hispanic and non-Hispanic patients, respectively. Mean change in SeBP in Hispanics ranged from −15.3/−7.3 mmHg for AML/OM 5/20 mg to −23.2/−13.8 mmHg for AML/OM 10/40 mg + hydrochlorothiazide 25 mg, and in non-Hispanics from −14.1/−7.8 mmHg to −25.4/−13.7 mmHg (all *p* < 0.0001 *versus* baseline). A majority of patients achieved mean 24 h, daytime, and nighttime ABP targets in both subgroups. Greater proportions of Hispanics achieved ABP targets *versus* non-Hispanics at week 12; however, this trend was reversed at week 20. Treatment was well tolerated.

**Conclusions:**

Switching to a fixed-dose combination of AML/OM ± hydrochlorothiazide provided significant BP lowering and effectively controlled BP in a large proportion of Hispanic and non-Hispanic patients with hypertension uncontrolled on previous monotherapy.

## Introduction

In the USA, the prevalence of hypertension and other risk factors for vascular disease is high in certain ethnicities and races, resulting in higher morbidity and mortality in these groups [Flack *et al*. 2010; Roger *et al*. 2011]. In Hispanic people, the prevalence of hypertension is lower than in black people, but the problems of undertreatment or inadequate treatment of hypertension and poor rates of blood pressure (BP) control leading to increased risk of cardiovascular events are shared by many ethnic or racial groups with hypertension [Egan *et al*. 2010; Roger *et al*. 2011]. A report from the National Health and Nutrition Examination Survey from 1999 to 2008 found that BP control rates have improved over recent years in white, black, and Hispanic groups, but awareness of hypertension (*p* = 0.03), treatment rates (*p* = 0.006), and BP control rates (*p* = 0.004) were all lower in Hispanic *versus* white people [Egan *et al*. 2010]. For Hispanics or Latinos, the following age-adjusted prevalence estimates from the National Health Interview Survey/National Center for Health Statistics were given for diagnosed conditions in people aged ≥18 years in 2009 [Pleis *et al*. 2010]: among Hispanics or Latinos, 21.5% had hypertension, 8.5% had heart disease, 5.8% had coronary heart disease, and 2.0% have had a stroke.

Current hypertension guidelines state that the majority of patients will require combination therapy with at least two antihypertensive medications to achieve BP control [Chobanian *et al*. 2003; Mancia *et al*. 2007, 2009]. In patients with hypertension, fixed-dose combination therapy with two or more medications can simplify the treatment regimen and potentially improve adherence [Bangalore *et al*. 2007].

Despite the availability of effective antihypertensive treatments and clear guidelines for the treatment of hypertension, ethnic minorities are often not treated appropriately to achieve BP goals; studies have shown that racial/ethnic differences in many aspects of hypertension are mainly due to social and environmental factors [Thorpe *et al*. 2008]. Barriers to achieving hypertension control in patients of all races include non-adherence to therapy, lack of awareness (though awareness is improving), lack of access to treatment, and clinical inertia (failure to intensify treatment) [Weinick *et al*. 2000; Okonofua *et al*. 2006; Yoon *et al*. 2010; Nelson *et al*. 2011].

The Blood Pressure Control in All Subgroups With Hypertension (BP-CRUSH) study evaluated improvement in BP goal achievement after patients whose BP was uncontrolled on previous antihypertensive monotherapy were switched to fixed-dose amlodipine/olmesartan medoxomil (AML/OM) with or without hydrochlorothiazide (HCTZ) combination therapy [Weir *et al*. 2011]. A subgroup analysis of BP reductions and goal achievement in Hispanic and non-Hispanic patients is reported here.

## Methods

### Study design

This was a prespecified subgroup analysis of Hispanic and non-Hispanic patients enrolled in the BP-CRUSH study [ClinicalTrials.gov identifier: NCT00791258], a phase IV (IIIb in South Africa), prospective, multicenter, open-label, single-arm, dose-titration study with a 20-week active treatment period. The results of the 20-week active treatment period, including study population demographics, inclusion and exclusion criteria, study design, efficacy and safety variables, and statistical analyses for the total cohort, are published elsewhere [Weir *et al*. 2011].

**Figure 1.**
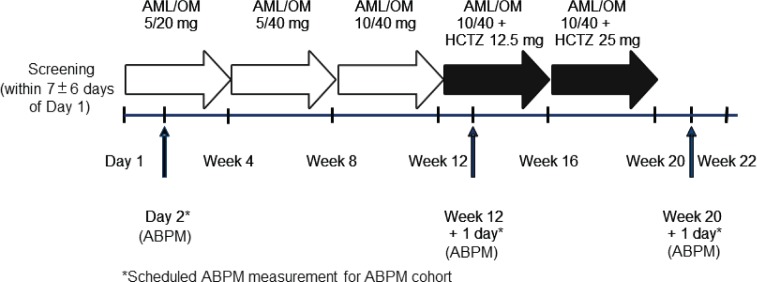
Study design. AML, amlodipine; ABPM, ambulatory blood pressure monitoring; HCTZ, hydrochlorothiazide; OM, olmesartan medoxomil. Reproduced with permission from Neutel J *et al*. (2012) *Adv Ther 29: 508–523*.

Patients with hypertension whose condition was uncontrolled on monotherapy after 1 month of treatment were screened for study eligibility. Full study inclusion/exclusion criteria have been described previously [Weir *et al*. 2011]. Briefly, the study inclusion criteria included uncontrolled BP [mean systolic BP (SBP) ≥140 mmHg (or ≥130 mmHg for patients with diabetes mellitus) and ≤180 mmHg and mean diastolic BP (DBP) ≤110 mmHg on two consecutive visits during screening] after at least 1 month of antihypertensive monotherapy with an angiotensin-converting enzyme inhibitor, angiotensin receptor blocker, β blocker, calcium channel blocker (CCB), or diuretic. On day 1, eligible patients were switched from their previous antihypertensive monotherapy to a fixed-dose combination of AML/OM 5/20 mg. Active treatment was administered once daily each morning. Uptitration was permitted every 4 weeks according to the following schedule (Figure 1): uptitration to AML/OM 5/40 mg, AML/OM 10/40 mg, AML/OM 10/40 mg + HCTZ 12.5 mg, and AML/OM 10/40 mg + HCTZ 25 mg. Patients were eligible to be uptitrated to any AML/OM combination dose if their mean SBP was at least 120 and less than 200 mmHg, or if their mean DBP was at least 70 and less than 115 mmHg. Patients were eligible to be uptitrated to any dose including an HCTZ add on if their mean SBP was at least 125 and less than 200 mmHg, or if their mean DBP was at least 75 and less than 115 mmHg. Patients with adequately controlled BP were maintained at their titrated drug dose level. However, if BP became uncontrolled during the maintenance phase (SBP ≥130 mmHg or DBP ≥80 mmHg), then patients were uptitrated to the next dosage level and re-entered the titration phase of the study. Patients receiving AML/OM 10/40 mg (±HCTZ) who achieved a mean SBP of less than 120 mmHg and a mean DBP of less than 70 mmHg, and who were asymptomatic for hypotension, entered maintenance treatment and continued in the study at their current dosage level. Patients with a mean SBP of at least 200 mmHg or DBP of at least 115 mmHg at any visit exited the study, as did patients with either an SBP of less than 120 mmHg or a DBP of less than 70 mmHg with symptomatic hypotension.

### Study assessments

The primary efficacy endpoint for the main study (and for this subgroup analysis) was the cumulative proportion of patients achieving the seated cuff SBP (SeSBP) goal of less than 140 mmHg (or <130 mmHg in patients with diabetes) during the first 12 weeks of active treatment.

Secondary endpoints included the proportion of patients achieving the SeSBP goal of less than 140 mmHg (or <130 mmHg in patients with diabetes) at the last post-baseline visit at or prior to week 12 [last observation carried forward (LOCF)]; the cumulative proportion of patients achieving the seated cuff BP (SeBP) goal of less than 140/90 mmHg (or <130/80 mmHg in patients with diabetes) from baseline to weeks 12 and 20; and the change from baseline in mean SeSBP and seated cuff DBP (SeDBP) by titration dose (LOCF). A cumulative approach to measuring the proportion of patients achieving the BP goal was used because BP is a dynamic variable and patients with hypertension achieve the BP goal at different time points during any clinical assessment period just as would be observed in a real-world setting. Consequently, this method was chosen rather than reporting BP goal rate for just one office visit which would not provide a comprehensive view of BP goal achievement over the course of treatment.

In the ambulatory BP (ABP) subgroup, endpoints included the proportion of patients achieving combined ABP monitoring (ABPM) targets at weeks 12 and 20, using the American Heart Association (AHA)-recommended values of less than 130/80 mmHg for mean 24 h ABP, less than 135/85 mmHg for mean daytime (8 am–4 pm) ABP, and less than 120/70 mmHg for mean nighttime ABP [Pickering *et al*. 2005], and the change from baseline in mean ambulatory SBP and DBP, including mean 24 h, daytime, and nighttime BP, and change in mean SBP and DBP during the last 2, 4, and 6 h of the dosing interval at weeks 12 and 20.

Safety assessments included the evaluation of adverse events, laboratory parameters, and physical examinations.

### Statistical analysis

The treated study population included all patients who received at least one dose of study medication. The ABPM subgroup included all treated patients with a valid baseline measurement and week 12 or 20 ABP data.

The cumulative SeBP goal achievement rate by visit was calculated as the ratio of the number of patients who achieved the goal at any time from the first dose date to the visit date over the number of patients who had any post-baseline BP data by that visit. These continuous efficacy variables were summarized with their corresponding 95% confidence intervals. Changes in SeBP and ABP from baseline were summarized by titration dose (LOCF method) and by visit without the LOCF method. The one-sample paired *t* test was used to test for significance, and all statistical analyses were performed at a two-sided significance level of 5%.

## Results

### Study disposition, demographics, and baseline characteristics

Out of a total of 1406 patients who were screened, 999 entered active treatment, of which 105 patients were of Hispanic ethnicity and 894 were non-Hispanic. These patients comprised the safety population (Table 1). The efficacy analysis population, defined as those patients who received at least one dose of the study drug and at least one post-baseline assessment, consisted of 100 Hispanic and 885 non-Hispanic patients. A total of 83 and 71 Hispanic patients had week 12 and week 20 SeBP measurements, respectively, compared with 782 and 674 for non-Hispanic patients. A total of 289 patients from the total population underwent ABPM, of which 44 patients were Hispanic and 245 were non-Hispanic. Changes from baseline and ABP target achievement were assessed in patients with baseline, week 12 and week 20 ABPM measurements.

**Table 1.** Demographics and baseline characteristics for Hispanic and non-Hispanic patients.

Characteristic	Hispanic (*n* = 105)	Non-Hispanic (*n* = 894)
Mean age, years (±SD)	52.9 (±11.15)	55.9 (±11.39)
≥65 years, *n* (%)	15 (14.3)	213 (23.8)
Women, *n* (%)	44 (41.9)	447 (50.0)
Weight (kg), mean (±SD)	86.31 (±16.47)	88.43 (±22.0)
Body mass index (kg/m^2^), mean (±SD)	30.91 (±4.38)	31.04 (±6.57)
Type 2 diabetes mellitus, *n* (%)	22 (21.0)	170 (19.0)
Metabolic syndrome,* *n* (%)	54 (51.4)	408 (45.6)
Glucose (mg/dl), mean (±SD)	107.93 (±23.748)	104.14 (±21.459)
HDL (mg/dl), mean (±SD)	50.62 (±13.514)	53.59 (±16.981)
Triglycerides (mg/dl), mean (±SD)	177.43 (±140.584)	154.16 (±93.735)
Cuff SBP (mmHg), mean (±SD)	153.59 (±9.10)	153.67 (±9.19)
Cuff DBP (mmHg), mean (±SD)	92.82 (±8.10)	91.81 (±8.67)
ABPM group, *n*	44	245
24 h ambulatory SBP (mmHg), mean (±SD)	135.49 (±12.61)	135.79 (±11.55)
24 h ambulatory DBP (mmHg), mean (±SD)	81.42 (±8.58)	81.03 (±9.49)

* Metabolic syndrome defined as the presence of at least three of the following: high-density lipoprotein (HDL) cholesterol <50 mg/dl in women and <40 mg/dl in men; triglycerides ≥150 mg/dl; blood pressure (BP) ≥130/85 mmHg; or fasting glucose ≥100 mg/dl. ABPM, ambulatory BP monitoring; DBP, diastolic BP; SBP, systolic BP; SD, standard deviation.

Several differences between Hispanic and non-Hispanic patients were evident at baseline. Hispanic patients were younger than non-Hispanic patients: mean age 52.9 *versus* 55.9 years, respectively. The proportion of patients aged at least 65 years was lower in the Hispanic cohort as well (14.3% *versus* 23.8%, respectively). The Hispanic cohort had a greater proportion of men than the non-Hispanic cohort (58.1% *versus* 50.0%, respectively), and more patients with metabolic syndrome (51.4% *versus* 45.6%).

Baseline BP (SeBP and ABP) was similar in both Hispanic and non-Hispanic patients. Baseline mean SeBP [±standard deviation (SD)] was 153.6 (±9.1)/92.8 (±8.1) mmHg and 153.7 (±9.2)/91.8 (±8.7) mmHg for Hispanic and non-Hispanic subgroups, respectively. Baseline mean 24 h ABP (±SD) was 135.5 (±12.6)/81.4 (±8.6) mmHg and 135.8 (±11.6)/81.0 (±9.5) mmHg, respectively.

### Efficacy

#### Cumulative systolic blood pressure goal achievement

**Figure 2.**
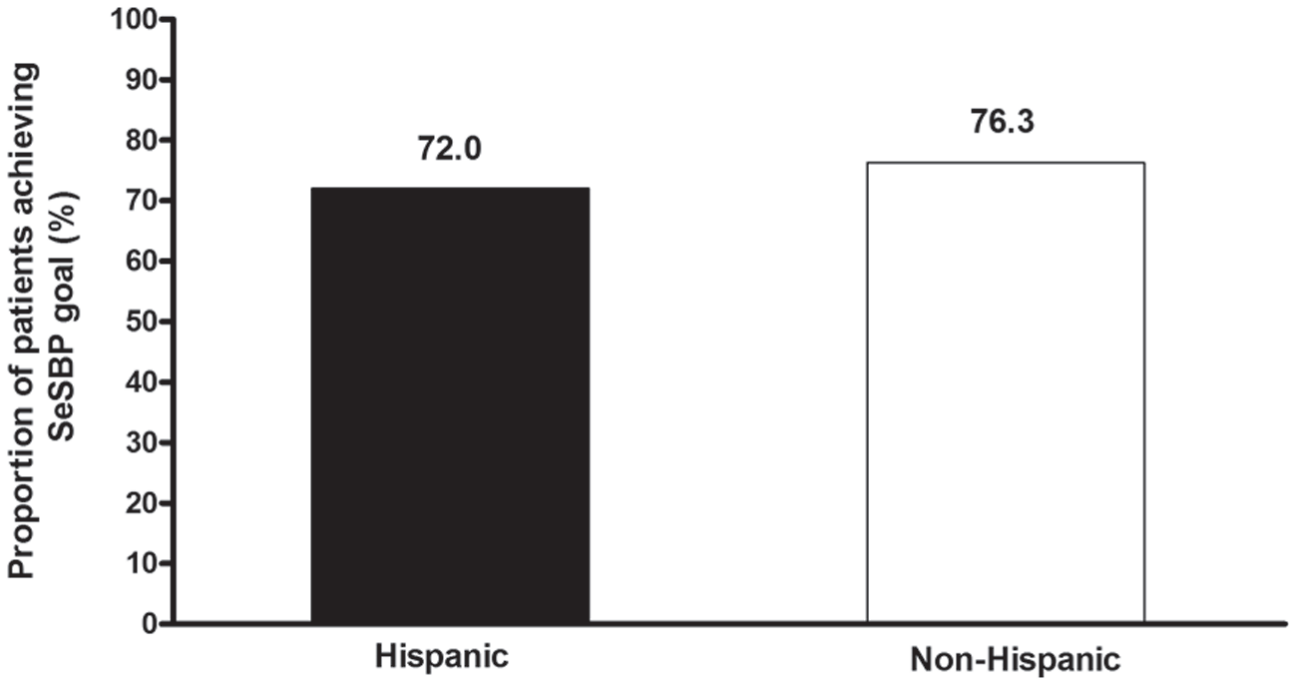
Cumulative proportions of Hispanic and non-Hispanic patients achieving the primary endpoint – week 12 seated cuff systolic blood pressure (SeSBP) goal of <140 mmHg (or <130 mmHg for patients with diabetes).

**Figure 3.**
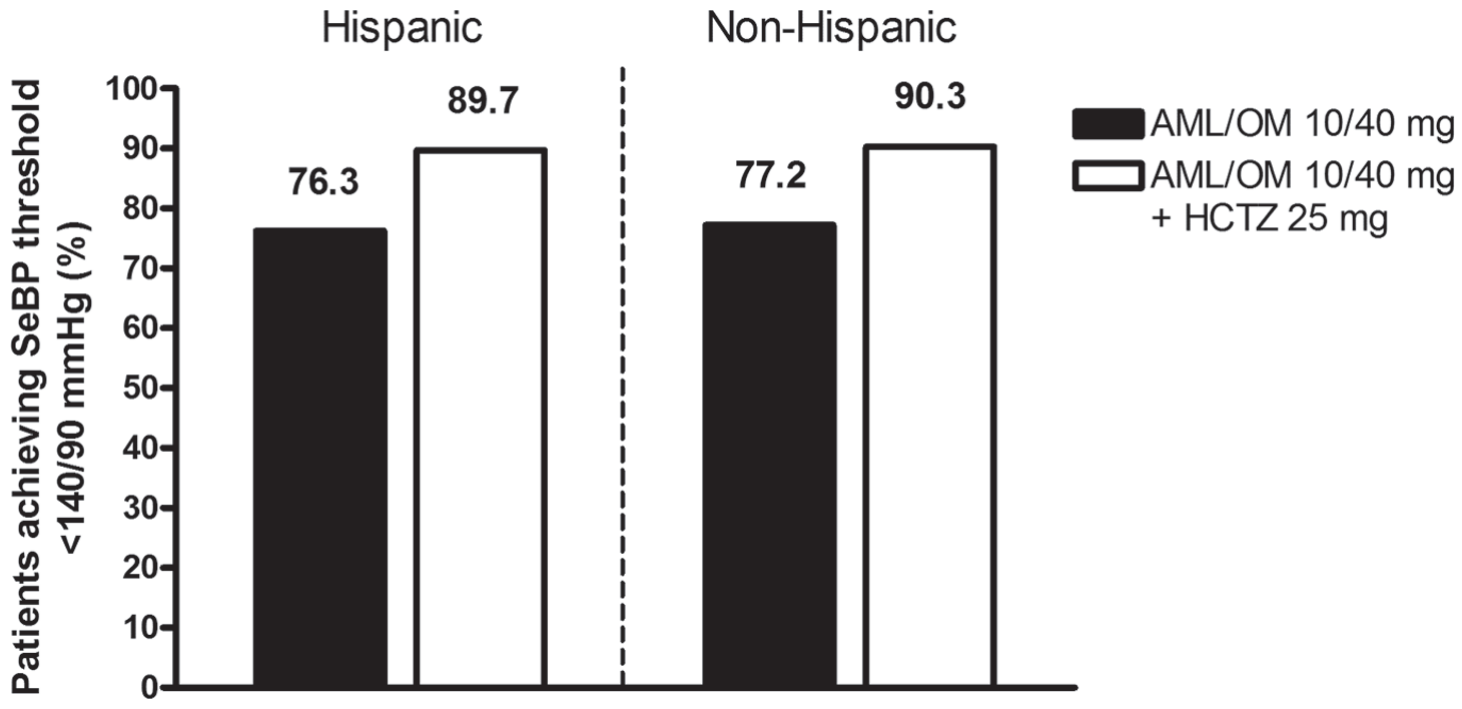
Cumulative proportion of patients achieving the seated cuff blood pressure (SeBP) threshold of <140/90 mmHg in the Hispanic and non-Hispanic subgroups at the highest dual combination (AML/OM 10/40 mg) and triple combination (AML/OM 10/40 mg + HCTZ 25 mg) therapy doses. AML, amlodipine; HCTZ, hydrochlorothiazide; OM, olmesartan medoxomil.

At the end of 12 weeks of active treatment, 72.0% of Hispanic patients and 76.3% of non-Hispanic patients achieved the primary endpoint of an SeSBP of less than 140 mmHg (or <130 mmHg for patients with diabetes) (Figure 2). Figure 3 shows the cumulative proportion of patients achieving the SeBP threshold of less than 140/90 mmHg at the highest dual combination (AML/OM 10/40 mg) and triple combination (AML/OM 10/40 mg + HCTZ 25 mg) therapy doses in the Hispanic and non-Hispanic subgroups. The proportions of patients achieving this BP threshold were similar for both Hispanic and non-Hispanic patients.

#### Reductions in seated cuff blood pressure

**Figure 4.**
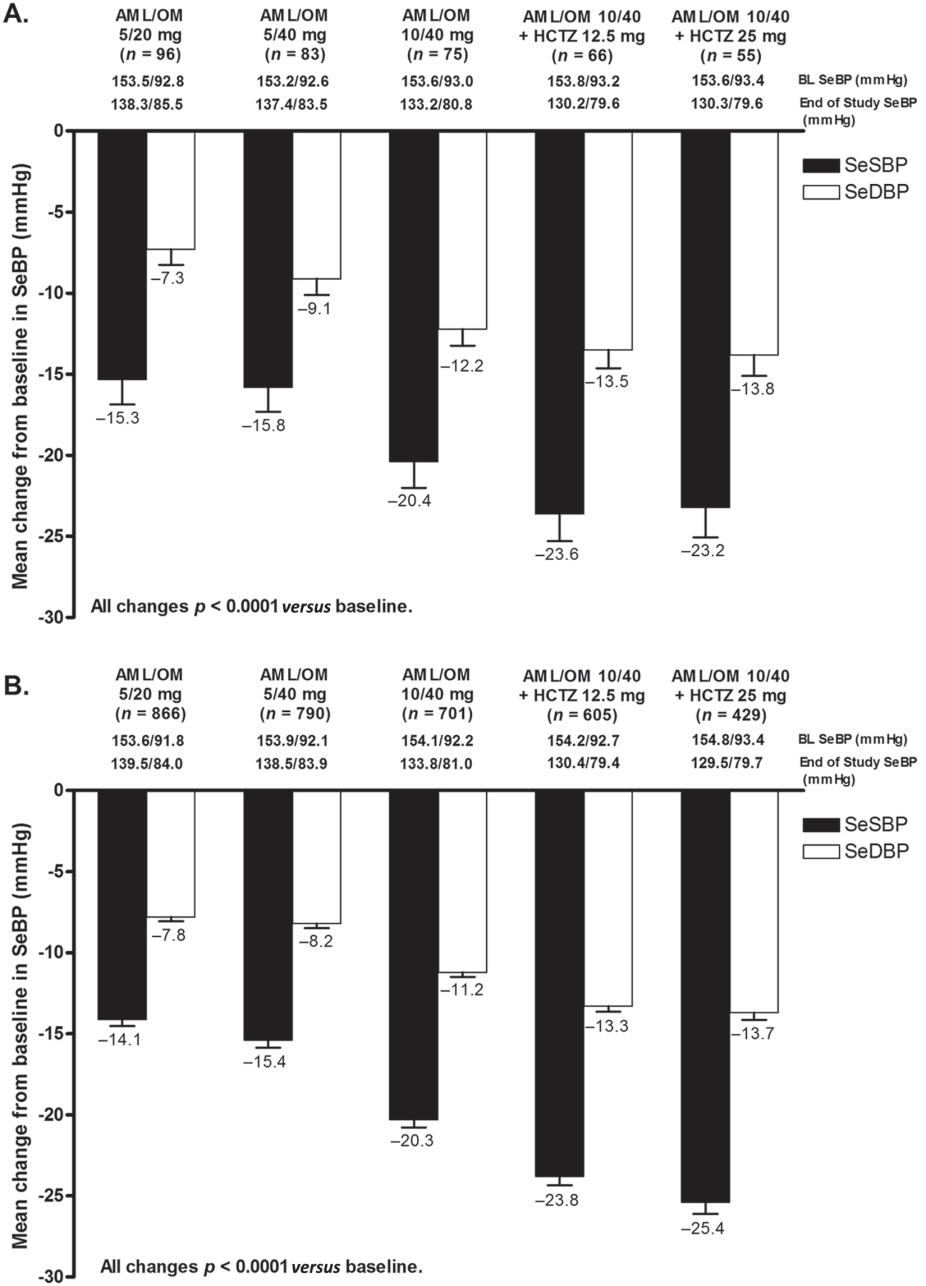
Change from baseline (BL) in seated cuff systolic and diastolic blood pressure (SeSBP and SeDBP) (±standard error of the mean) by titration dose (last observation carried forward) in (A) Hispanic and (B) non-Hispanic patients. AML, amlodipine; HCTZ, hydrochlorothiazide; OM, olmesartan medoxomil; SeBP, seated cuff blood pressure.

Mean SeSBP was significantly reduced from baseline at the week 12 and week 20 visits in the Hispanic (22.4 and 25.2 mmHg, respectively; both *p* < 0.0001) and non-Hispanic (21.7 and 26.9 mmHg; both *p* < 0.0001) subgroups. BP lowering achieved with AML/OM-based therapy was similar in both Hispanic (Figure 4A) and non-Hispanic (Figure 4B) subgroups. SeSBP and SeDBP were significantly reduced from baseline at the end of each titration dose period (LOCF) in Hispanic and non-Hispanic patients (*p* < 0.0001). Mean [±standard error of the mean (SEM)] (LOCF) changes from baseline in SeSBP/SeDBP during the titration periods ranged from −15.3 (±1.6)/−7.3 (±1.0) mmHg at the AML/OM 5/20 mg dose to −23.2 (±1.8)/−13.8 (±1.3) mmHg at the AML/OM 10/40 mg + HCTZ 25 mg dose for Hispanic patients and from −14.1 (±0.4)/−7.8 (±0.3) mmHg to −25.4 (±0.7)/−13.7 (±0.4) mmHg, respectively, for non-Hispanic patients.

#### Ambulatory blood pressure target achievement

**Figure 5.**
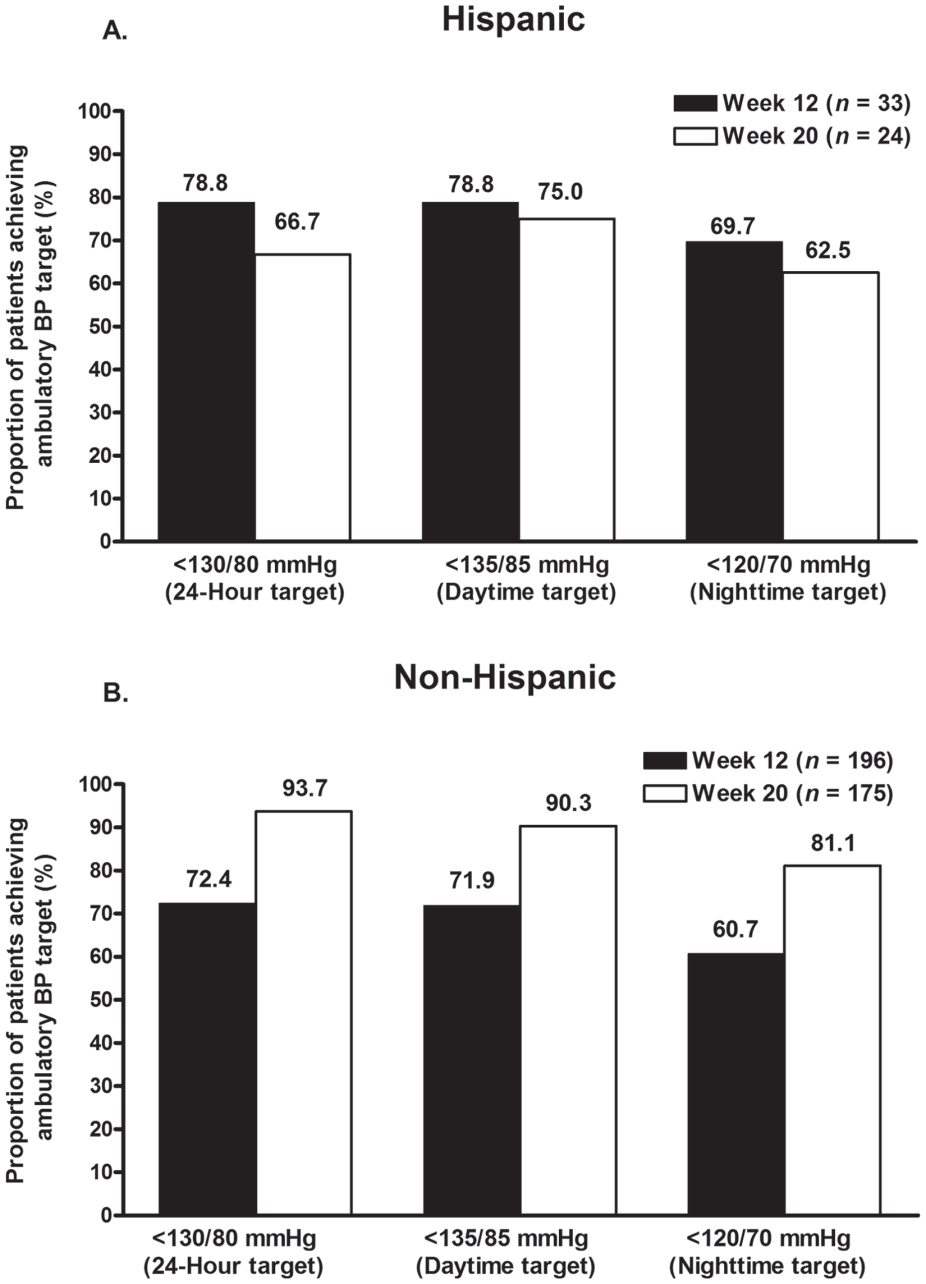
Proportions of (A) Hispanic and (B) non-Hispanic patients achieving mean 24 h (<130/80 mmHg), daytime (8 am–4 pm) (<135/85 mmHg), and nighttime (10 pm–6 am) (<120/70 mmHg) ambulatory blood pressure (BP) targets at weeks 12 and 20.

In Hispanic and non-Hispanic patients who underwent ABPM, the proportions of patients achieving ABP targets based on AHA-recommended normal values are shown in Figure 5.

A majority of patients attained mean 24 h, daytime, and nighttime ABP targets in both cohorts. However, a reduction in ABP target rates was observed from week 12 to week 20 in Hispanic patients. At weeks 12 and 20, 78.8% and 66.7% of Hispanic patients, respectively, achieved a mean 24 h ABP of less than 130/80 mmHg; 78.8% and 75.0% of Hispanic patients achieved a mean daytime ABP value of less than 135/85 mmHg; and 69.7% and 62.5% of Hispanic patients achieved a mean nighttime ABP value of less than 120/70 mmHg (Figure 5A). Non-Hispanic patients showed an increase in ABP target achievement from week 12 to week 20. At weeks 12 and 20, 72.4% and 93.7% of non-Hispanic patients, respectively, achieved a mean 24 h ABP of less than 130/80 mmHg; 71.9% and 90.3% achieved a mean daytime ABP value of less than 135/85 mmHg; and 60.7% and 81.1% achieved a mean nighttime ABP value of less than 120/70 mmHg (Figure 5B). A greater proportion of Hispanic patients achieved all three ABP targets at 12 weeks compared with non-Hispanic patients; however, a greater proportion of non-Hispanic patients achieved all three ABP targets at 20 weeks compared with Hispanic patients. In addition, the proportions of Hispanic patients attaining these same ABP targets were observed to decrease from week 12 to week 20.

#### Reductions in mean ambulatory blood pressure

**Figure 6.**
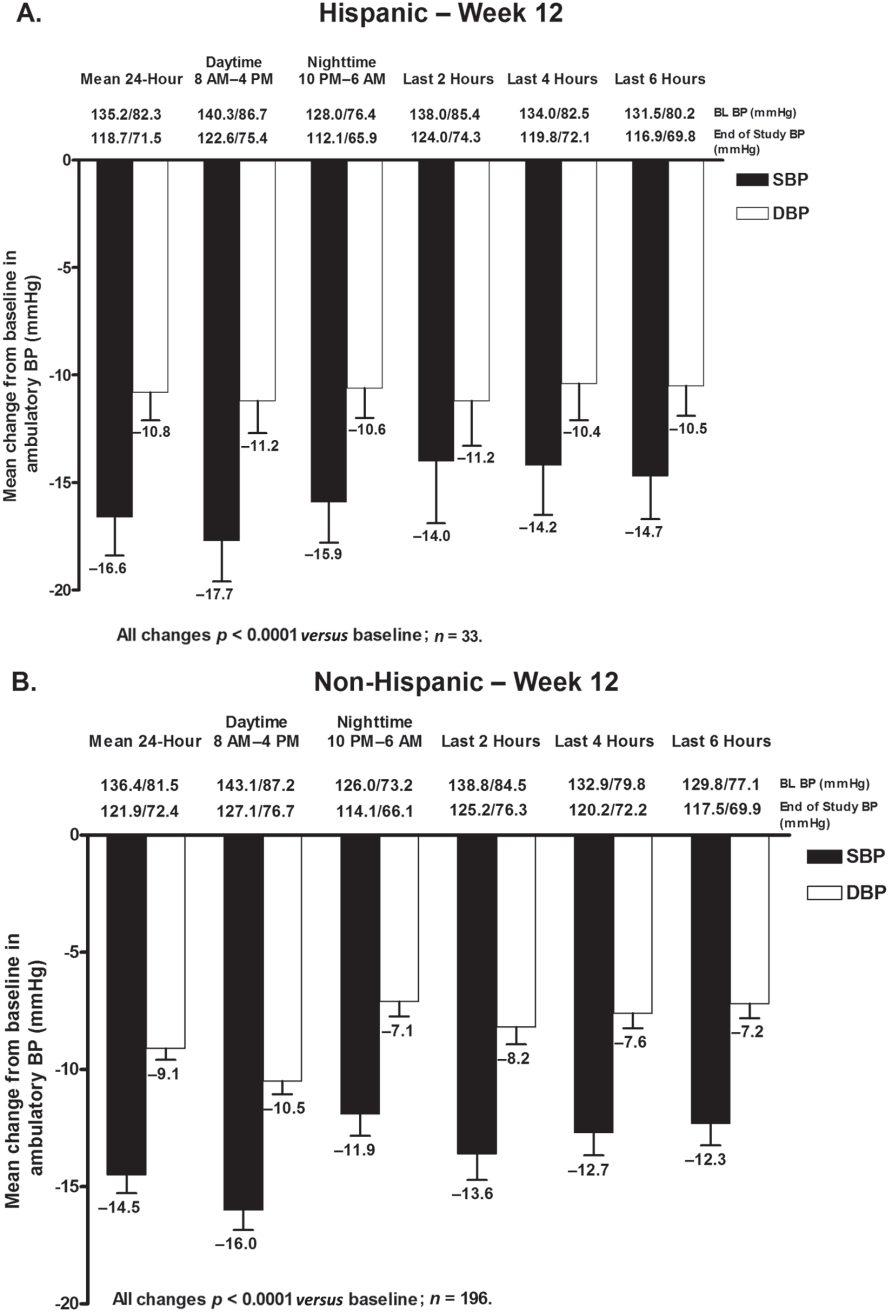
Change in mean ambulatory systolic BP (SBP) and diastolic BP (DBP) (±standard error of the mean) during the 24 h dosing interval, daytime (8 am–4 pm), nighttime (10 pm–6 am), and the last 2, 4, or 6 h of the dosing interval at week 12 in (A) Hispanic and (B) non-Hispanic patients. BL, baseline; BP, blood pressure.

**Figure 7.**
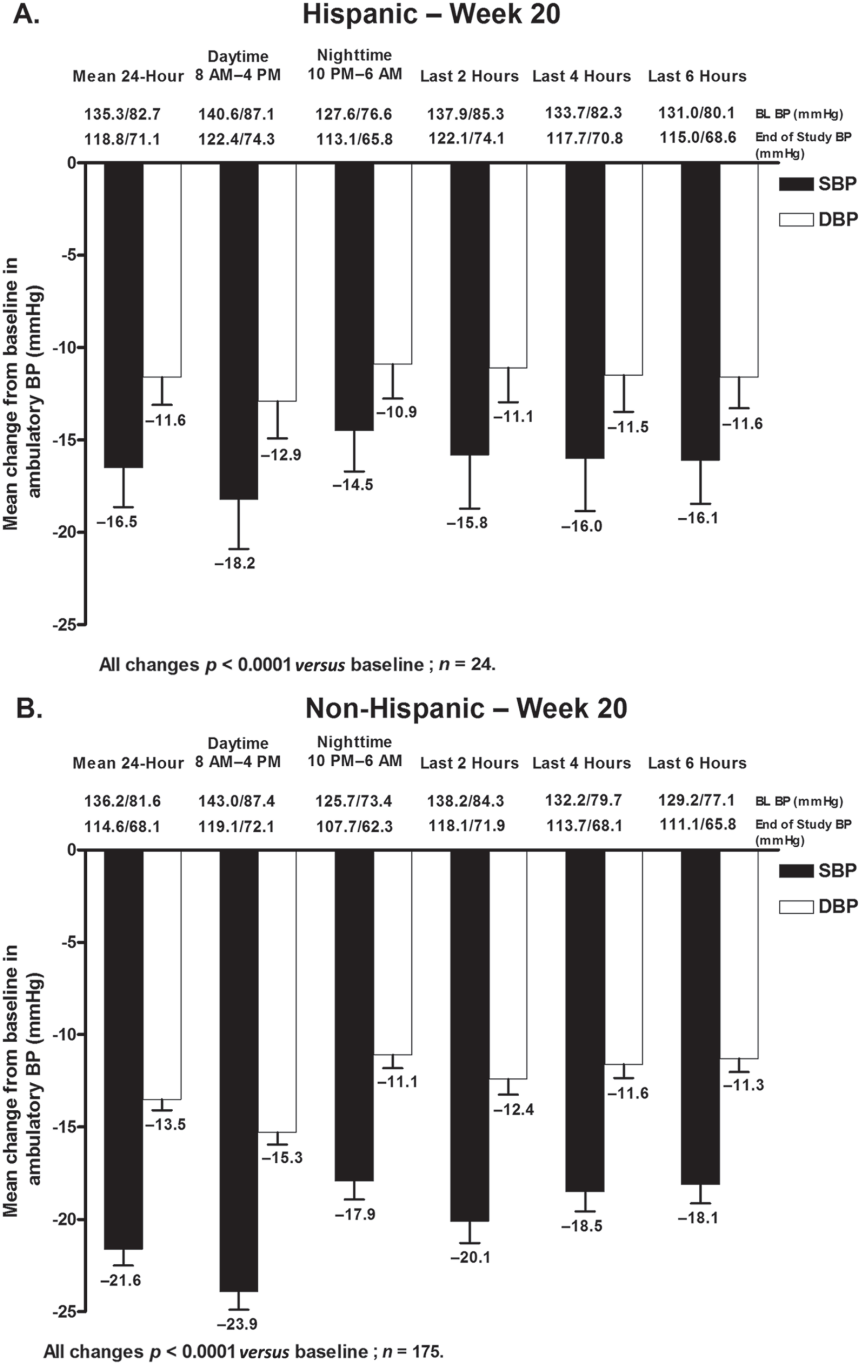
Change in mean ambulatory systolic BP (SBP) and diastolic BP (DBP) (±standard error of the mean) during the 24 h dosing interval, daytime (8 am–4 pm), nighttime (10 pm–6 am), and the last 2, 4, or 6 h of the dosing interval at week 20 in (A) Hispanic and (B) non-Hispanic patients. BL, baseline; BP, blood pressure.

Mean 24 h, daytime, and nighttime ambulatory SBP and DBP were all significantly decreased from baseline at week 12 and week 20 in both Hispanic and non-Hispanic subgroups (Figures 6 and 7). The change in mean 24 h ABP (±SEM) at week 12 (Figure 6) and week 20 (Figure 7) was −16.6 (±1.8)/−10.8 (±1.3) mmHg and −16.5 (±2.1)/−11.6 (±1.5) mmHg in Hispanic patients and −14.5 (±0.8)/−9.1 (±0.5) mmHg and −21.6 (±0.9)/−13.5 (±0.6) mmHg in non-Hispanic patients (Figures 6 and 7).

Mean changes in ambulatory SBP and DBP in the last 2, 4, and 6 h of the dosing interval were also all significantly decreased from baseline at week 12 in Hispanic and non-Hispanic patients (Figure 6). Similar results were observed at week 20 (Figure 7). For all ABP time points at week 12, SBP and DBP reductions were greater in Hispanic patients compared with non-Hispanic patients. Conversely, with the exception of DBP during the last 6 h, SBP and DBP reductions were greater in non-Hispanic patients *versus* Hispanic patients at week 20.

### Safety and tolerability

Overall, the treatment regimen was well tolerated in both Hispanic and non-Hispanic subgroups. The proportion of Hispanic and non-Hispanic patients experiencing any treatment-emergent adverse event (TEAE) was 39.0% (*n* = 41) and 54.6% (*n* = 488), and the proportion experiencing a drug-related TEAE was 20.0% (*n* = 21) and 26.2% (*n* = 234), respectively (Table 2). The majority of TEAEs were mild to moderate in severity, and the proportion of patients who experienced a TEAE decreased with the addition of HCTZ to AML/OM treatment. No serious adverse events (SAEs) were reported in Hispanic patients, whereas 1.3% (*n* = 12) of non-Hispanic patients reported an SAE. None of these SAEs were considered to be drug related.

**Table 2.** Summary of treatment-emergent adverse events.

AE, *n* (%)	Hispanic patients		Non-Hispanic patients	
	AML/OM (*n* = 105)	AML/OM + HCTZ (*n* = 68)	AML/OM (*n* = 894)	AML/OM + HCTZ (*n* = 632)
Patients with any TEAE^*^	37 (35.2)	14 (20.6)	392 (43.8)	225 (35.6)
Patients with any drug-related TEAE	16 (15.2)	8 (11.8)	154 (17.2)	100 (15.8)
Patients with any serious TEAE	0 (0.0)	0 (0.0)	8 (0.9)	4 (0.6)
Patients with any SAE	0 (0.0)	0 (0.0)	8 (0.9)	4 (0.6)
Patients with any drug-related SAE	0 (0.0)	0 (0.0)	0 (0.0)	0 (0.0)
Patients with any TEAE leading to discontinuation	4 (3.8)	1 (1.5)	49 (5.5)	31 (4.9)
Patients with any drug-related TEAE leading to discontinuation	4 (3.8)	1 (1.5)	38 (4.3)	26 (4.1)
Drug-related TEAEs in ≥1% of patients				
Peripheral edema	7 (6.7)	2 (2.9)	48 (5.4)	9 (1.4)
Dizziness	1 (1.0)	4 (5.9)	38 (4.3)	35 (5.5)
Headache	1 (1.0)	0 (0.0)	15 (1.7)	4 (0.6)
Fatigue	1 (1.0)	0 (0.0)	13 (1.5)	6 (0.9)
Hypotension	2 (1.9)	0 (0.0)	12 (1.3)	9 (1.4)
Nausea	1 (1.0)	0 (0.0)	9 (1.0)	4 (0.6)
Blood uric acid increased	0 (0.0)	0 (0.0)	0 (0.0)	8 (1.3)

* A treatment-emergent adverse event (TEAE) is any AE that either first occurred on/after the first active dose date, or occurred before the first active dose date, then occurred again during active treatment with worsened severity, and occurred no later than 14 days after the last active dose. AE, adverse event; AML, amlodipine; HCTZ, hydrochlorothiazide; OM, olmesartan medoxomil; SAE, serious AE.

A total of 4.8% (*n* = 5) of Hispanic patients and 7.2% (*n* = 64) of non-Hispanic patients discontinued from the study due to a drug-related TEAE. No deaths due to an adverse event were reported. The most frequently reported drug-related TEAEs in Hispanic and non-Hispanic patients were peripheral edema and dizziness. Peripheral edema occurred in 7.6% (*n* = 8) of Hispanic patients and in 6.4% (*n* = 57) of non-Hispanic patients, while dizziness occurred in 4.8% (*n* = 5) of Hispanic patients and in 7.9% (*n* = 71) of non-Hispanic patients. The incidence of drug-related hypotension was similar and low in both the Hispanic and non-Hispanic subgroups, that is, 1.9% (*n* = 2) and 2.3% (*n* = 21), respectively.

The incidence of drug-related peripheral edema decreased with the addition of HCTZ in both the Hispanic (from 6.7% to 2.9%) and non-Hispanic (from 5.4% to 1.4%) subgroups.

## Discussion

The results from this subgroup analysis of the BP-CRUSH study demonstrate that an AML/OM-based titration regimen was a well tolerated means of effectively reducing BP and achieving SeBP goals and ABP targets in Hispanic and non-Hispanic patients with hypertension uncontrolled on previous antihypertensive monotherapy. The majority of patients achieved the primary endpoint of a cuff SBP of less than 140 mmHg (or <130 mmHg in patients with diabetes) at week 12 on an AML/OM-based combination therapy regimen. Combined SeBP goal rates (<140/90 or <130/80 mmHg) were also achieved in a majority of patients at weeks 12 and 20. SeBP goal rates at both time points were slightly lower in Hispanic patients (69.0% and 81.0%) *versus* non-Hispanic patients (71.5% and 85.2%).

An effective antihypertensive agent should not only reduce BP but should also provide BP control throughout the dosing interval. Data have shown that the morning surge in BP resulting from variations in the circadian rhythm of BP is associated with an increased risk of cardiovascular events, particularly during the first 2 h after awakening [Gosse *et al*. 2004]. Thus, agents that provide 24 h BP control play an important role in potentially reducing cardiovascular events and improving outcomes. Although there are no guideline-approved ABP targets, the suggested AHA 24 h, daytime, and nighttime ABP targets (<130/80 mmHg, <135/85 mmHg, and <120/70 mmHg, respectively) were achieved at weeks 12 and 20 in a majority of Hispanic and non-Hispanic patients in this subgroup analysis. Mean 24 h, daytime, and nighttime ABP target rates achieved at week 12 were similar in Hispanic and non-Hispanic patients, with higher proportions of Hispanic patients achieving these same targets; however, greater proportions of non-Hispanic patients had achieved these ABP targets compared with the Hispanic subgroup at week 20. It should be noted that the number of Hispanic patients who underwent ABPM was small at 44. This may be one possible explanation for the discrepancy observed in week 12 and week 20 ABP target achievement rates in Hispanic patients. Overall, mean SeBP and mean 24 h, daytime, and nighttime ABP, and ABP measured in the last 2, 4, and 6 h of the dosing interval were all significantly reduced from baseline at weeks 12 and 20 in all patients.

Fixed-dose combination therapy with AML/OM, with or without HCTZ, was well tolerated in this patient population; no new safety issues were observed. The incidence of peripheral edema that has been previously associated with CCB therapy was expectedly highest in both the Hispanic and non-Hispanic groups for the AML/OM combination. The addition of HCTZ decreased its incidence in both cohorts, which was also reflected in the decreased incidence of drug-related TEAEs reported for the AML/OM + HCTZ treatment group. Thus, these data support previous observations that the incorporation of a thiazide diuretic into a high-dose AML regimen can help to reduce the incidence of edema. Although not subject to statistical analysis, the incidence of drug-related TEAEs appeared to be lower in Hispanic patients compared with the non-Hispanic population. Taken together, these data support the utility of an AML/OM-based titration regimen in providing well tolerated, 24 h BP control in Hispanic patients with hypertension uncontrolled on previous monotherapy.

Although guidelines advise that the use of at least two antihypertensive agents will be necessary for most patients to achieve BP goals in the seventh report of the Joint National Committee on Prevention, Detection, Evaluation, and Treatment of High Blood Pressure and in European guidelines [Chobanian *et al*. 2003; Mancia *et al*. 2007, 2009], this aspect of the guidelines is often not well translated into clinical practice [Spranger *et al*. 2004]. In addition to lack of awareness, lack of access to treatment, and clinical inertia [Cushman and Basile, 2006; Basile and Neutel, 2010], a main barrier to BP goal achievement is patient non-adherence to treatment, which is known to be associated with increasing pill burden [Bangalore *et al*. 2007]. The development of fixed-dose combinations such as AML/OM help decrease pill burden, thereby potentially increasing patient adherence to treatment and the likelihood of BP goal achievement [Bangalore *et al*. 2007].

Treating to BP goal is important for all patients, regardless of their ethnicity/race, in order to minimize adverse cardiovascular events [Chobanian *et al*. 2003]. Analyses such as ours demonstrate that high rates of BP control are achievable in Hispanic patients; however, the difficulties lie in translating goal rates achieved in clinical trials to the real-world clinical setting [Singer *et al*. 2002; Cushman and Basile, 2006]. Treat-to-goal strategies for hypertension management and initiatives to improve awareness and treatment of hypertension have been shown to reduce the ethnic/racial disparities in hypertension control by eliminating some of the previously discussed barriers to BP control [Singer *et al*. 2002; Manze *et al*. 2010; Hebert *et al*. 2011].

Limitations of this subgroup analysis include the open-label, single-arm design, possibly resulting in treatment bias due to lack of blinding. For the ABP data analysis, the number of patients in the Hispanic subgroup was relatively small, and thus these results need to be assessed with caution if extrapolating to similar populations seen in the clinical setting due to low statistical power. In addition, potential gender effects on antihypertensive efficacy were not assessed in this study and it would be interesting to evaluate the effect of gender in combination with ethnicity in future studies. The strengths of this subgroup analysis were that it was prespecified in the study protocol and that it provides efficacy and safety data in a subgroup of patients that is often under-reported in the literature.

## Conclusions

This prespecified subgroup analysis showed that switching to a fixed-dose combination of AML/OM ± HCTZ provided significant BP lowering and effectively controlled BP to achieve SeSBP and SeBP targets, as well as mean 24 h, daytime, and nighttime ABP targets in a large proportion of both Hispanic and non-Hispanic patients with hypertension whose condition was previously uncontrolled on antihypertensive monotherapy. In addition to achieving SeBP goals and 24 h ABP targets, the AML/OM ± HCTZ combination therapy regimen was well tolerated in both Hispanic and non-Hispanic patients.
